# Incidence rates of narcolepsy diagnoses in Taiwan, Canada, and Europe: The use of statistical simulation to evaluate methods for the rapid assessment of potential safety issues on a population level in the SOMNIA study

**DOI:** 10.1371/journal.pone.0204799

**Published:** 2018-10-17

**Authors:** Caitlin N. Dodd, Maria de Ridder, Wan-Ting Huang, Daniel Weibel, Maria Giner-Soriano, Silvia Perez-Vilar, Javier Diez-Domingo, Lawrence W. Svenson, Salahddin M. Mahmud, Bruce Carleton, Monika Naus, Jeffrey C. Kwong, Brian J. Murray, Lisen Arnheim-Dahlstrom, Lars Pedersen, Rosa Morros, Francisco Javier Puertas, Steven Black, Miriam Sturkenboom

**Affiliations:** 1 Erasmus Medical Center, Rotterdam, The Netherlands; 2 Julius Center Global Health, University Medical Center Utrecht, Utrecht, The Netherlands; 3 Taiwan Centers for Disease Control, Taipei, Taiwan; 4 Institut Universitari d’Investigació en Atenció Primària Jordi Gol (IDIAP Jordi Gol), Barcelona, Spain; 5 Universitat Autònoma de Barcelona, Bellaterra (Cerdanyola del Vallès), Barcelona, Spain; 6 Fundación para el Fomento de la Investigación Sanitaria y Biomédica de la Comunitat (FISABIO), Vaccine Research, Valencia, Spain; 7 University of Alberta, Division of Preventative Medicine, Alberta, Canada; 8 University of Manitoba, Rady Faculty of Health Sciences, Winnipeg, Manitoba, Canada; 9 University of British Columbia, Faculty of Medicine, Vancouver, British Columbia, Canada; 10 Institute for Clinical Evaluative Sciences, Toronto, Ontario, Canada; 11 Public Health Ontario, Toronto, Ontario, Canada; 12 Department of Family & Community Medicine, University of Toronto, Toronto, Ontario, Canada; 13 Dalla Lana School of Public Health, University of Toronto, Toronto, Ontario, Canada; 14 University Health Network, Toronto, Ontario, Canada; 15 Division of Neurology, Department of Medicine, Sunnybrook Health Sciences Centre, Toronto, Canada; 16 Karolinska Institut, Department of Medical Epidemiology and Biostatistics, Stockholm, Sweden; 17 Aarhus University, Department of Clinical Epidemiology, Aarhus, Denmark; 18 Sleep Unit, Neurophysiology Department, La Ribera University Hospital, Valencia, Spain; 19 Physiology Department, University of Valencia, Valencia, Spain; 20 Cincinnati Children’s Hospital, Center for Global Health, Cincinnati, Ohio, United States of America; Associazione OASI Maria SS, ITALY

## Abstract

**Background & objectives:**

Vaccine safety signals require investigation, which may be done rapidly at the population level using ecological studies, before embarking on hypothesis-testing studies. Incidence rates were used to assess a signal of narcolepsy following AS03-adjuvanted monovalent pandemic H1N1 (pH1N1) influenza vaccination among children and adolescents in Sweden and Finland in 2010. We explored the utility of ecological data to assess incidence of narcolepsy following exposure to pandemic H1N1 virus or vaccination in 10 sites that used different vaccines, adjuvants, and had varying vaccine coverage.

**Methods:**

We calculated incidence rates of diagnosed narcolepsy for periods defined by influenza virus circulation and vaccination campaign dates, and used Poisson regression to estimate incidence rate ratios (IRRs) comparing the periods during which wild-type virus circulated and after the start of vaccination campaigns vs. the period prior to pH1N1 virus circulation. We used electronic health care data from Sweden, Denmark, the United Kingdom, Canada (3 provinces), Taiwan, Netherlands, and Spain (2 regions) from 2003 to 2013. We investigated interactions between age group and adjuvant in European sites and conducted a simulation study to investigate how vaccine coverage, age, and the interval from onset to diagnosis may impact the ability to detect safety signals.

**Results:**

Incidence rates of narcolepsy varied by age, continent, and period. Only in Taiwan and Sweden were significant time-period-by-age-group interactions observed. Associations were found for children in Taiwan (following pH1N1 virus circulation) and Sweden (following vaccination). Simulations showed that the individual-level relative risk of narcolepsy was underestimated using ecological methods comparing post- vs. pre-vaccination periods; this effect was attenuated with higher vaccine coverage and a shorter interval from disease onset to diagnosis.

**Conclusions:**

Ecological methods can be useful for vaccine safety assessment but the results are influenced by diagnostic delay and vaccine coverage. Because ecological methods assess risk at the population level, these methods should be treated as signal-generating methods and drawing conclusions regarding individual-level risk should be avoided.

## Introduction

In August 2010, a safety signal of narcolepsy following AS03-adjuvanted pdm(09)H1N1 influenza vaccine Pandemrix® was reported in Finland and Sweden among children and adolescents [1]. Other rapid risk assessment studies conducted in the European Union (EU) did not show changes in incidence rates of narcolepsy diagnoses, except in Finland, Sweden, and Norway [2], all countries that achieved high coverage rates with Pandemrix. Subsequent hypothesis-testing studies showed associations; these had high within- and between-study variation [3]. In China, where vaccine coverage was very low, a 3-fold increase in narcolepsy onset was reported following the peak of the pandemic [4].

Narcolepsy is a rare disease with a long interval from onset of symptoms to diagnosis, especially in adults. Several possible explanations for the purported pdm(09)H1N1 and narcolepsy link have been proposed but none confirmed. Hypotheses range from a causal effect of the AS03 adjuvant, the manufacturing process, presence of nucleoproteins in Pandemrix, and molecular mimicry, to awareness and assessment biases, and residual confounding [5–10]. Based upon simulation studies conducted by Wijnans et al., in the absence of a causal association but in the presence of accelerated diagnosis due to awareness, we would expect to see an increased incidence of narcolepsy diagnosis following awareness of the association followed by a decrease, even to levels below the background incidence, due to depletion of cases[8]. This effect may be particularly important in conditions with a long delay to diagnosis such as in narcolepsy where the delay in diagnosis from initial symptoms can be 10–20 years [11].

The SOMNIA (Systematic Observational Method for Narcolepsy and Influenza Immunization Assessment) study was funded by the US Centers for Disease Control and Prevention (CDC) and used information from countries that used different types of adjuvanted pandemic influenza vaccines to assess whether the pdm(09)H1N1 influenza vaccine and specifically the MF59 and AS03 adjuvants were associated with narcolepsy.

One of the goals of SOMNIA was to assess patterns of incidence rates of narcolepsy in multiple geographic areas and to understand changes in incidence rates of narcolepsy diagnoses before, during, and after the pdm(09)H1N1 influenza pandemic by using electronic health care data, which may be rapidly available. In this paper, we explore whether assessment of safety signals based on ecological methods and population-based electronic health care data are suitable for vaccine safety risk assessment, by exploiting the heterogeneity in vaccine coverage, types of vaccines, and vaccination programs across countries. We assess what strength of signals can be detected using population-level data collected before and after a hypothetical targeted vaccination campaign.

Ecological studies can be defined as those that measure exposure and outcomes at the group level rather than at the individual level [12, 13]. In such a study, groups are defined by a naturally occurring difference in space or time such as a change in the vaccination schedule [14] or the beginning and end of a targeted vaccination campaign [15].

This study may serve as an example of the utility of ecological methods to assess vaccine safety signals, particularly regarding events with long onset-to-diagnosis intervals.

## Materials and methods

Narcolepsy diagnosis incidence rates were evaluated in ten sites representing seven countries spanning three continents (Taiwan (TW), Canada (CA) [Manitoba, Alberta, and British Columbia], The Netherlands (NL), The United Kingdom (UK), Sweden (SE), Denmark (DK), and Spain (ES) [Valencia and Cataluña]) using population-based electronic healthcare databases originating from general practitioners (GPs) (UK, ES, NL) or claims/record linkage databases (SE, DK, TW [16–18], and CA) (S1 Table).

### Study population and follow-up

For data sources in which individual linkage can be made between population and diagnoses (all sites except Sweden and British Columbia, Canada), the study population comprised all individuals registered within each of the databases during the study period. Observation time began on the date of first registration of an individual in the database, the start of the study period (January 2003), or the start date of data collection for the database, whichever was the latest and ended on the date of death, the date registration was terminated, the end of data collection, or the end of the study period (December 2013), whichever was the earliest. Sweden and British Columbia, Canada used census data to calculate person-time denominators. We used a harmonized approach in which databases locally extracted their data into simple input files in a common format that could be locally analyzed and aggregated using SAS or JAVA-based software [2, 19].

### Case identification and validation

Cases were persons with a new diagnosis of narcolepsy with or without cataplexy. Validation of the diagnostic codes using patient discharge letters and medical records was conducted in the GP databases in the Netherlands and Valencia, Spain. For these two sites, only validated cases were used in the analysis. The other sites used algorithms combining diagnostic codes for narcolepsy with claims for multiple sleep latency tests (MSLTs) to reduce the false positive rate. The same method was used at each site over the entire time period. No further validation was done in other sites (S1 Table).

### Analysis

To investigate the purported narcolepsy-pandemic vaccine effect, incidence rates of narcolepsy diagnosis were calculated by calendar year and month and also categorized into three periods based on specific circulation/vaccination periods in each country: 1) pre-pandemic (from January 2003 until the start of the period of pH1N1 circulation); 2) during pH1N1 wild-type virus circulation until the start of the country’s pH1N1 vaccination campaign; and 3) from the start of the pH1N1 influenza vaccination campaign through the end of the study (S1 Table). Pandemic H1N1 virus circulation was defined as the period during which weekly influenza test positivity for pH1N1 infection exceeded 10%. Dynamic age groups were categorized as <5 years, 5–19 years, 20–59 years, and ≥60 years at the time of diagnosis. This categorization was motivated by differences in diagnosis for each age group, and particularly the challenges of differential diagnosis in young children and the elderly [20, 21]. Incidence rates of narcolepsy diagnoses were calculated by dividing the number of narcolepsy cases by the accumulated person-time. Ninety-five percent confidence intervals (CIs) were calculated assuming a negative binomial distribution. Following confirmation of homogeneity in incidence rates among databases within the same country, further analyses were conducted at the level of the country rather than the site.

Within each country, we estimated incidence rate ratios (IRRs) and 95% CIs for each time period using Poisson regression, with the pre-circulation period as a reference. We included terms for age strata, time periods, and an age*time period interaction using time periods as defined by pH1N1 circulation and vaccination campaign dates.

We conducted additional analyses restricted to European countries to estimate the impact of vaccine coverage and adjuvant among children and adolescents and separately among adults. For this analysis, a composite variable summarizing vaccine coverage classified as low (<20%) or high (≥20%) and adjuvant (MF59 or AS03) was created, and incidence in the period after vaccination had started was compared to the pre-pH1N1 circulation period. Because the composite adjuvant/coverage variable was collinear with database and country, neither database nor country was included in the European model.

### Simulation

To better understand the utility of ecological methods for assessing vaccine safety signals and whether an association in one age group may be masked in a population-level analysis, we conducted statistical simulations. Each set consisted of 10,000 subjects aged 0 to 100 years, who were observed in the period January 1, 2003 to December 31, 2012. The period of the vaccination campaign was set from October to December 2009. In each simulated data set, vaccine coverage during this period was set at one out of 9 different values between 1 and 99 percent (see Table 1). Subjects assigned as vaccinated got a vaccination date during the campaign period. Baseline narcolepsy incidence was simulated based on reported estimates of incidence by decade of age [22].For vaccinated persons aged <20 years, during the six months following vaccination, the risk for narcolepsy onset was simulated using a relative risk varying from 0.5 to 10 compared to the baseline incidence. In subjects aged ≥20 years no increased risk was applied. The median time from onset to diagnosis was initially set at 4 years for adults and at 1.5 years for children (aged ≤18 years) based on SOMNIA data (not shown). The effect of the length of the interval from onset to diagnosis was also tested by varying a reduction rate parameter, with 0 removing the interval (i.e. immediate diagnosis following onset), 0.5 halving the interval, and 1 retaining the full simulated interval (see Table 1 for description of simulation parameters). Finally, in part of the sets the full 10 years was used as study period, in the others it was restricted to 6 months after the end of the vaccination campaign (7.5 years).

**Table 1:** Simulation parameters.

Parameter	Definition	Levels
Vaccine Coverage	Probability of vaccination	.01, .05, .10. .25, .50, .75, .95, .99
Relative Risk	Relative risk of narcolepsy onset in the first 6 months after vaccination	0.5, 1, 2, 3, 4, 5, 6, 7, 8, 9, 10
Interval Reduction Rate	Reduction of onset-to-diagnosis interval	0: Immediate diagnosis following onset
		0.5: Halving of the onset-to-diagnosis interval
		1: Full onset-to-diagnosis interval
Observation Length	Total length of observation time following start of observation in January 2003	2739 days: pre-vaccination campaign time + 90 days vaccination campaign + 6 month risk period
		3625 days: 10 years

For each set of simulation parameters, 500 replications were run. The population-level incidence rate ratio for the period following the vaccination campaign vs. the period prior to the vaccination campaign was estimated using Poisson regression. The median incidence rate ratio from the 500 replications was calculated overall and by age group, and plotted against vaccine coverage. The percentage bias was calculated by subtracting the simulated relative risk from the estimated IRR and dividing by the simulated IRR.

### Calibration

Using the simulation results, a model was constructed to determine how the underlying individual-level relative risk was related to the median estimated IRR and the appointed vaccine coverage and interval reduction, and the interaction of these terms (Model: Simulated individual-level true RR = Estimated IRR + Simulated vaccine coverage + Simulated onset-to-diagnosis reduction + Interactions). This model was then applied to results obtained from the observed data, using the IRR found in the SOMNIA study and the relevant vaccine coverage and diagnostic delay (S1 Table), in order to calculate the underlying relative risks in 5- to 19-year olds, that would have been necessary to produce this IRR in the absence of other sources of bias. This calibration was restricted to the 5–19 year age group as this group was the source of the safety signal and the only subjects for whom an increased risk was simulated.

This study was conducted under the principles of the Helsinki declaration and each site was responsible for obtaining appropriate ethical approvals. The overall study was approved by the Institutional Review Board at Cincinnati Children’s Hospital, Cincinnati, Ohio, USA. The study was also approved at each site by the following bodies: Health Research Ethics Board, Alberta, Canada; University of Manitoba Research Ethics Board, Manitoba, Canada; Ethics Committee for Clinical Research of the Directorate General of Public Health and Center for Advance Research in Public Health (CEIC DGSP/CSISP), Valencia, Spain; Comitè Ètic d'Investigació de l'IDIAP Jordi Gol, Cataluña, Spain; Regional Ethics Review Board, Stockholm, Sweden; The Institutional Review Board of Centers for Disease Control, Ministry of Health and Welfare, Taiwan; THIN Scientific Review Committee, United Kingdom; Erasmus Medical Center Medical Internal Review Board, The Netherlands. Due to use of aggregate data, British Columbia, Canada and Denmark conducted the study under rolling approval and study-specific approval was not sought.

## Results

### Observed incidence

Incidence rates of narcolepsy diagnoses ranged from 0.22 to 1.52 per 100,000 person-years by site (Table 2). Incidence rates in databases within the same country (Canada and Spain) were similar so for further analysis country-specific data were pooled.

**Table 2:** Crude incidence rates by site.

Site	Period	Events	Person-years	IR
**EU**				
Denmark	2003–2013	269	17,850,129	1.50 (1.33–1.69)
United Kingdom	2003–2013	467	42,897,721	1.09 (0.99–1.19)
ES, Valencia (validated)	2009–2013	46	20,458,082	0.22 (0.17–0.28)
ES, Cataluña	2007–2013	240	34,861,809	0.69 (0.50–0.78)
Sweden	2003–2013	1536	102,027,209	1.52 (1.43–1.59)
The Netherlands (validated)	2003–2013	14	2,879,712	0.49 (0.29–0.76)
**North America**				
CA, British Columbia	2003–2013	278	47,857,684	0.58 (0.32–0.64)
CA, Alberta	2003–2013	427	51,885,946	0.82 (0.74–0.90)
CA, Manitoba	2003–2010	42	6,335,257	0.66 (0.50–0.86)
**Asia**				
Taiwan	2003–2012	472	161,407,503	0.29 (0.27–0.32)

Abbreviations: IR (Incidence Rate), EU (European Union), ES (Spain), CA (Canada)

**Fig 1:**
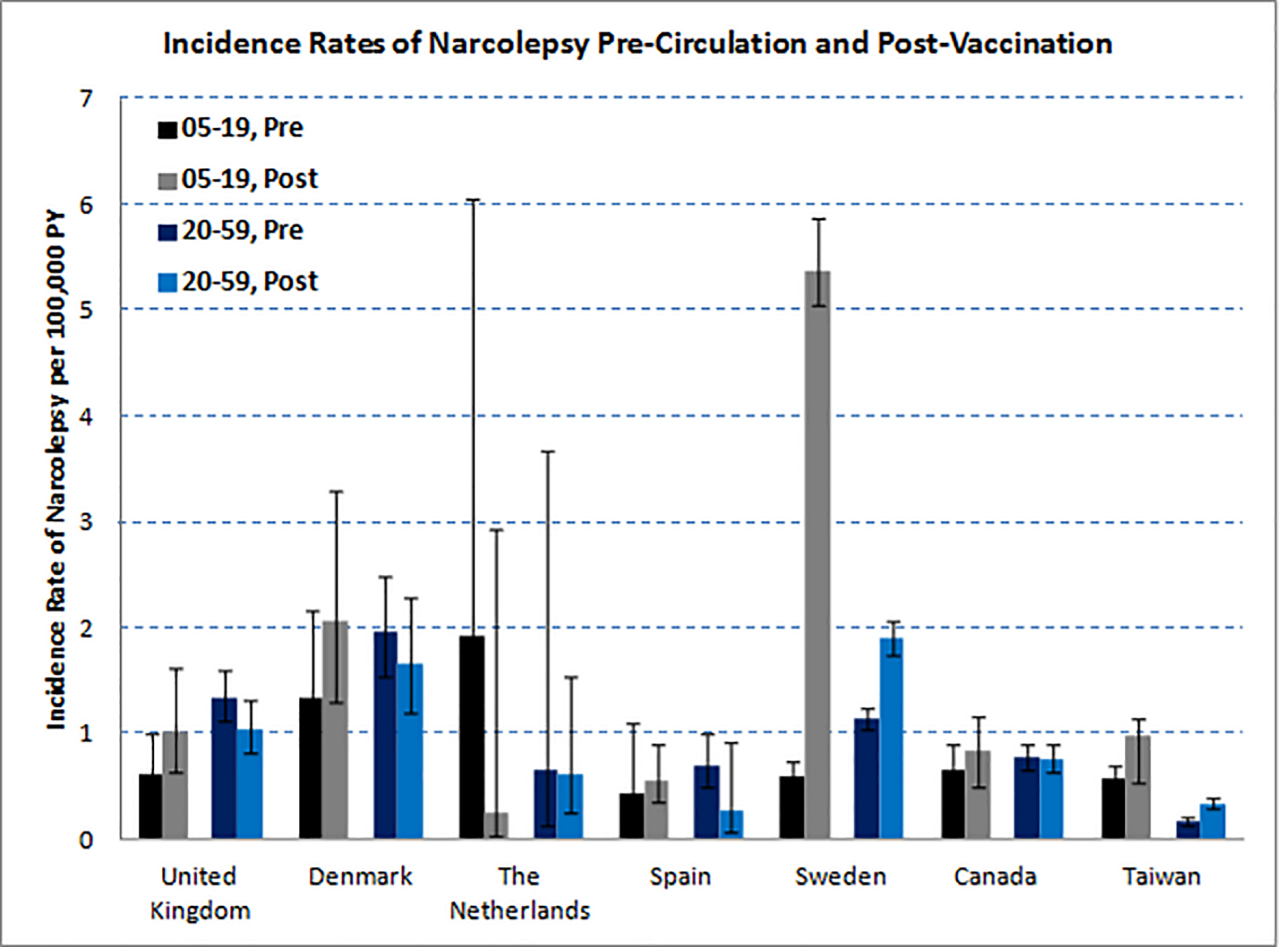
Incidence rates (with 95% CIs) of narcolepsy pre-circulation and post-vaccination in children (05–19 yrs.) and adults (20–59 yrs.).

Due to very low rates observed among the very young (<5 years) and the elderly (≥60 years) (S2 Table) and known difficulties in diagnosis in these age groups, these were not included in further analyses. In Fig 1, IRs are shown stratified by age group and time period. In investigation of age group and time period and the interaction of these factors, IRRs were significantly elevated in both age groups in Taiwan (where MF59-adjuvanted vaccine coverage was 59% for those <19 years and 11% for those ≥19 years) in the period during circulation of wild-type virus prior to vaccination. For those aged 5–19 years, the IRR was 2.50 (95%CI 1.46, 4.28), and for those aged 20–59 years, the IRR was 2.23 (95%CI 1.26, 3.94). This continued in the period after the vaccination campaign had started, with IRR 1.60 (95%CI 1.20, 2.13) for those aged 5–19 years and IRR 2.13 (95%CI 1.62, 2.79) for those aged 20–59 years (Table 3). In Sweden, where AS03-adjuvanted Pandemrix vaccine coverage was 60%, in the period after vaccination incidence rates among those aged 5–19 years and 20–59 years were elevated [IRR = 9.01 (95%CI 6.89, 11.80) and IRR = 1.69 (95%CI 1.46, 1.95)], respectively (Fig 1 & Table 3). None of the other countries showed significant time-period-by-age-group interactions.

**Table 3:** IRs and IRRs by country, age and period.

Site	Age	Period*	Cases	Person years	IR	IRR†	95%CI
United Kingdom	5–19	Pre-Circulation	26	4247239	0.61	Ref	—
		Circulation	0	229303	0.00	NA	—
		Vaccination & Post	28	2752486	1.02	1.66	0.97, 2.83
	20–59	Pre-Circulation	183	13782669	1.33	Ref	—
		Circulation	9	744620	1.21	0.91	0.47, 1.78
		Vaccination & Post	90	8706262	1.03	0.78	0.61, 1.00
Denmark	5–19	Pre-Circulation	26	1941950	1.34	Ref	—
		Circulation	3	160562	1.87	1.40	0.42, 4.61
		Vaccination & Post	28	1352428	2.07	1.55	0.91, 2.64
	20–59	Pre-Circulation	103	5258884	1.96	Ref	—
		Circulation	8	416864	1.92	0.98	0.48, 2.01
		Vaccination & Post	58	3492758	1.66	0.85	0.62, 1.17
The Netherlands	5–19	Pre-Circulation	2	103950	1.92	Ref	
		Circulation	0	29453	0.00	NA	
		Vaccination & Post	1	394895	0.25	0.13	0.01, 1.45
	20–59	Pre-Circulation	2	306773	0.65	Ref	
		Circulation	1	87315	1.15	1.76	0.16, 19.37
		Vaccination & Post	7	1144346	0.61	0.94	0.20, 4.52
Spain	5–19	Pre-Circulation	7	1617473	0.43	Ref	
		Circulation	4	1488771	0.27	0.62	0.18, 1.13
		Vaccination & Post	26	4715178	0.55	1.27	0.55, 2.94
	20–59	Pre-Circulation	48	6847254	0.70	Ref	—
		Circulation	33	610444	0.54	0.77	0.50, 1.20
		Vaccination & Post	125	18915104	0.27	0.94	0.68, 1.31
Sweden	5–19	Pre-Circulation	62	10381883	0.60	Ref	—
		Circulation	1	819877	0.12	0.20	0.03, 1.47
		Vaccination & Post	369	6854603	5.38	9.01	6.89, 11.80
	20–59	Pre-Circulation	338	29823712	1.13	Ref	—
		Circulation	26	2418238	1.08	0.95	0.64, 1.41
		Vaccination & Post	401	20992445	1.91	1.69	1.46, 1.95
Canada	5–19	Pre-Circulation	67	10107116	0.66	Ref	—
		Circulation	6	1261204	0.48	0.72	0.31, 1.70
		Vaccination & Post	53	6378494	0.83	1.25	0.87, 1.80
	20–59	Pre-Circulation	265	34413993	0.77	Ref	—
		Circulation	36	4574717	0.79	1.02	0.72, 1.45
		Vaccination & Post	182	24228401	0.75	0.98	0.81, 1.18
Taiwan	5–19	Pre-Circulation	81	13985353	0.58	Ref	—
		Circulation	16	1103680	1.45	2.50	1.46, 4.28
		Vaccination & Post	110	11867183	0.93	1.60	1.20, 2.13
	20–59	Pre-Circulation	78	46806947	0.17	Ref	—
		Circulation	14	3768896	0.37	2.23	1.26, 3.94
		Vaccination & Post	158	44542437	0.35	2.13	1.62, 2.79

*Periods are as follows: Pre-Circulation = January 2003-the beginning of wild-type H1N1 circulation (defined per country); Circulation = Period from the beginning of wild-type H1N1 circulation until the start of the vaccination campaign (defined per country); Vaccination & Post = Period from the beginning of the vaccination campaign through December 2013.

† IRR comparing the period to the pre-circulation period, within the age group

In the analysis restricted to Europe and including a vaccine coverage/adjuvant composite variable, IRRs were elevated in the period following start of vaccination in the high-coverage AS03 (Sweden) and low-coverage AS03 groups for children and adolescents (S3 Table). In adults, an elevated incidence in the period following vaccination was detected in the AS03 high-coverage group, which was limited to Sweden. In this analysis, no changes in the incidence of narcolepsy in the post-vaccination period were seen in sites using MF59-adjuvanted vaccine, all of which had low coverage (S3 Table).

### Simulation

**Fig 2:**
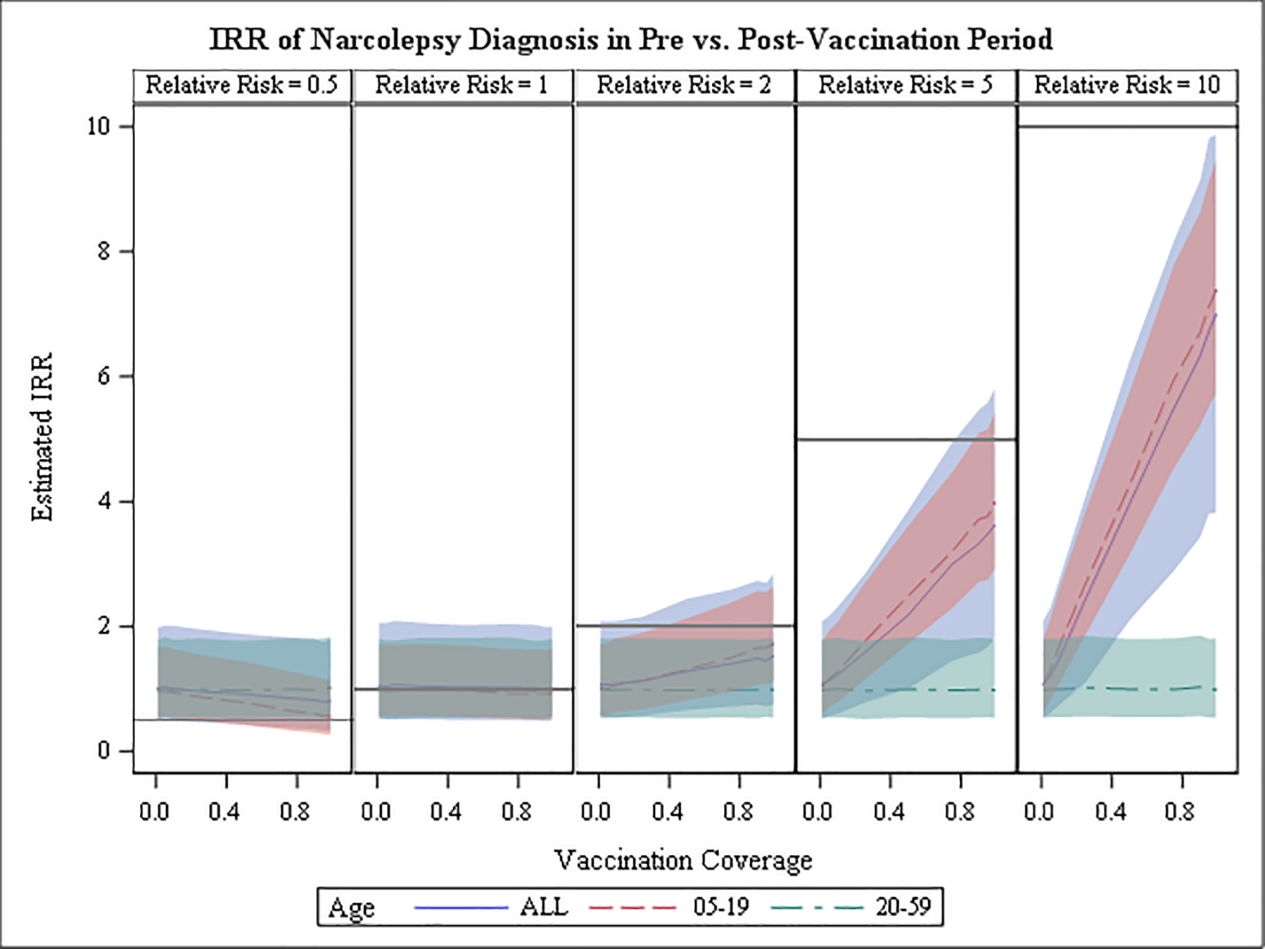
IRR estimates in simulated data, immediate diagnosis (interval reduction rate = 0). Population-level incidence rate ratio estimated from simulated data with observation time equal to 3625 days and true individual-level relative risk equal to .05, 1, 2, 5, or 10 (columns). Gray horizontal reference lines represent the true simulated individual-level relative risk of narcolepsy diagnosis. The interval reduction rate parameter is set equal to zero (meaning immediate diagnosis following onset of symptoms). Vaccination coverage increases within each column along the x-axis. Colored lines represent age group-specific IRRs as noted in the legend and colored bands represent associated 95% confidence intervals.

**Fig 3:**
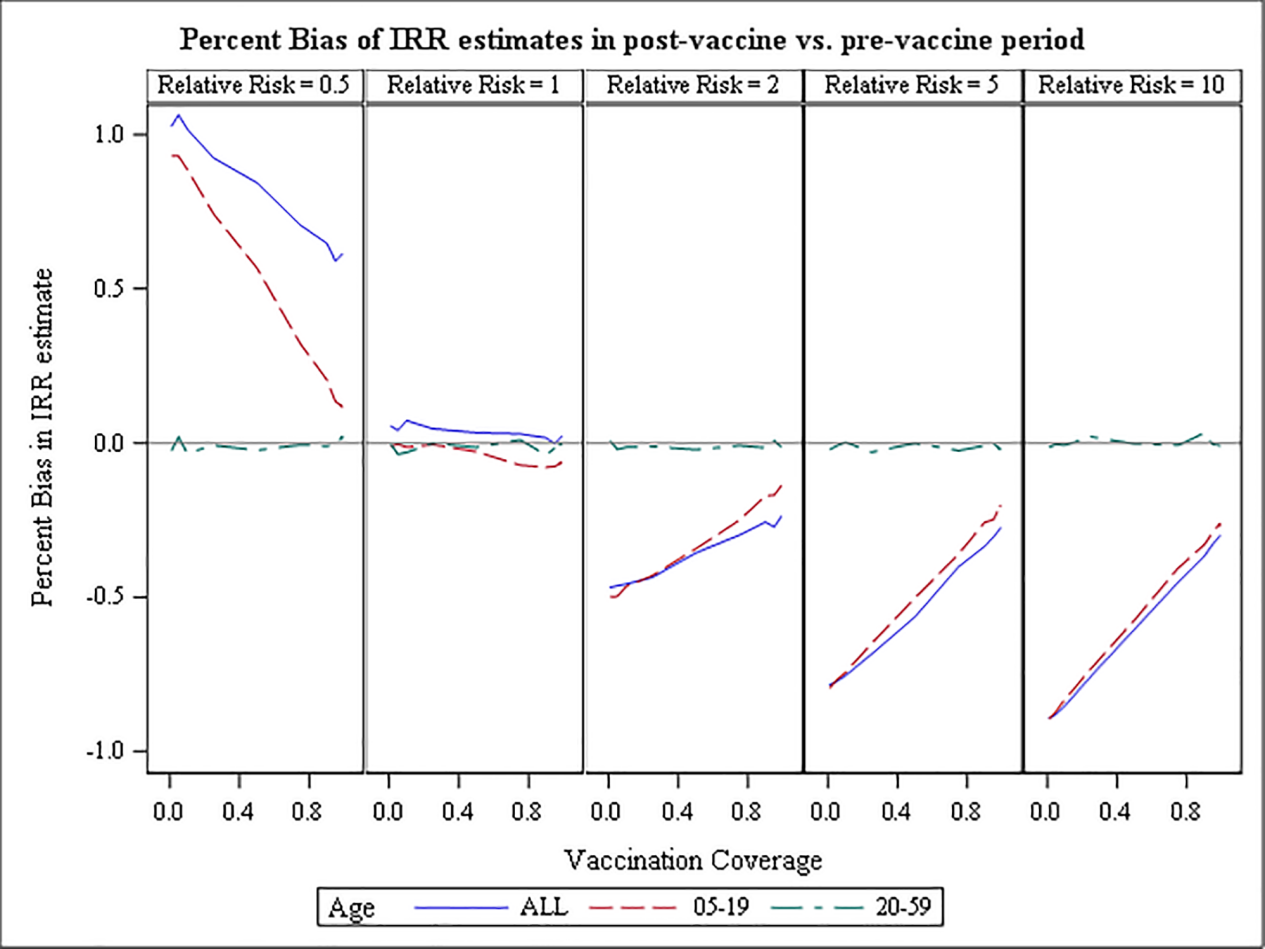
Percent bias of IRR estimates in simulated data, immediate diagnosis (interval reduction rate = 0). Population-level percent bias estimated from simulated data with observation time equal to 3625 days and true individual-level relative risk equal to .05, 1, 2, 5, or 10 (columns). The (interval reduction rate parameter is set equal to zero (meaning immediate diagnosis following onset of symptoms). Vaccination coverage increases within each column along the x-axis. Colored lines represent age group-specific bias as noted in the legend.

The simulation study showed that in an analysis such as the one described above, the true RR is consistently underestimated when it is greater than one and overestimated when less than one (Fig 2). Underestimation of true relative risks greater than one is attenuated as vaccination coverage increases but the estimate remains about 6%-26% too low even with vaccination coverage as high as 99% with no delay from onset to diagnosis and a 10-year observation period (Fig 3). As the interval from onset to diagnosis increases, the estimated relative risk deviates further from the true relative risk; performance is improved if the observation time captures only the period of increased risk (data not shown). When the time from onset to diagnosis was not reduced no increased risk was detected for any set of simulation parameters (data not shown). Stratification by age group was effective in elucidating the group that was the source of the increased risk.

### Calibration

When using a model derived from the results of the simulation study to predict which should have been the true (calibrated) IRR to produce the estimates found in the SOMNIA study, these true IRRs were always considerably higher than the estimates. Notably, according to the model, the required individual-level relative risk underlying a 9-fold IRR which was found in 5–19 year olds in Sweden should have been 36.04 (95% CI: 27.79, 46.90) (Table 4).

**Table 4:** IRRs calibrated using model derived from simulated data.

Country	Age Group	Observed Ecological IRR	95% CI	Gamma Scale	Vaccine Coverage	Calibrated IRR	95% CI
UK	5–19	1.66	0.97, 2.83	1	.05	7.76	4.95, 12.53
Denmark	5–19	1.55	0.91, 2.64	1	.05	7.31	4.71, 11.76
Netherlands	5–19	0.13	0.01, 1.45	1	.1	1.64	1.16, 6.84
Spain	5–19	1.27	0.55, 2.94	1	.05	6.17	3.24, 12.98
Sweden	5–19	9.01	6.89, 11.80	0.5	.12	36.04	27.79, 46.90
Canada	5–19	1.25	0.87, 1.80	1	.35	5.87	4.62, 7.68
Taiwan	5–19	1.60	1.20, 2.13	0	.65	2.75	1.75, 4.07

## Discussion

Evaluation of incidence rates on a population level can be done relatively quickly in countries/regions with accessible population-based electronic health care databases. This is useful for assessing potential vaccine safety signals. In order to calculate rates quickly in a standardized manner, harmonization of data into simple input files in a common format allowed for the pooling and sharing of data across three continents. The method was capable of identifying the signal in Sweden in 5- to 19-year olds.

In the analysis by country, elevated rates of narcolepsy were only detected in Taiwan during wild-type virus circulation through the period following vaccination with MF59-adjuvanted and non-adjuvanted vaccines, and in Sweden following vaccination with AS03-adjuvanted vaccines. The finding in Taiwan may be due to circulation of wild-type influenza virus prior to the start of the vaccination campaign [17]. This is consistent with the finding of a 3-fold increase in narcolepsy onset in China following the peak of the pH1N1 pandemic in a population with very low vaccine coverage [4]. Taiwan vaccinated children aged <1 year with MF59-adjuvanted vaccine and adults and school children with mainly non-adjuvanted vaccine. In Sweden, where the signal of a narcolepsy safety concern was originally detected [23] and where patients diagnosed with narcolepsy are being compensated [7], rates were much higher than in the other countries. This could be due to differential reporting due to increased awareness of the putative association, a true causal effect in this population with this vaccine, or some combination of these factors. As shown in simulations, reduction in the time from onset to diagnosis due to awareness of an association can lead to artificial inflations in risk estimates [8]. In Canadian provinces, with around 40% vaccine coverage of a different AS03-adjuvanted vaccine (Arepanrix^TM^), no effect was seen in any of the age groups or periods. This study, which is by necessity observational, has several limitations. Data were collected according to a shared protocol but using locally derived algorithms, which may have led to differences in sensitivity and specificity. Case validation in some sites revealed low specificity of the original extraction, which may be the case in other sites as well. In our analysis by adjuvant and vaccine coverage, high coverage with AS03-containing vaccine was only present in Sweden, making Sweden and this adjuvant/coverage group collinear. This makes it impossible to determine whether we are seeing the effects of the vaccine itself or of the reporting and detection patterns in each country. Additionally, the manufacturing process of Arepanrix^TM^ differed from that of Pandemrix^TM^, leading to vaccines containing different quantities of influenza virus components [24]. The potential effects of these differences in manufacturing cannot be differentiated from adjuvant specific-effects or from other country-specific effects using an ecological design such as the one presented here. Similarly, the countries in which MF59 was used were the same countries in which case validation was conducted. This limits comparability between these countries and others and, therefore, between MF59-containing vaccines and other pandemic vaccines.

Differences in case ascertainment could also have impacted our estimates. For example, it has been noted elsewhere that the safety signal originated in Sweden and that this, together with compensation for cases, may have impacted diagnosis patterns [6, 7]. Additionally, due to the healthcare system in Taiwan, children complaining of excessive daytime sleepiness are seen by a specialist quickly, making the interval from onset to diagnosis for these children shorter. A median time from symptom onset to MSLT referral of 60 days has been reported for pediatric narcolepsy cases in Taiwan[25]. While it is not possible to rule out a causal association, it is important to note that these factors undoubtedly contributed to the estimates obtained in this study. Differences in the prevalence of the underlying HLA-DQB1*06:*02* risk allele for narcolepsy, which has been reported to vary widely by country, may affect the incidence at the population level but is unlikely to have affected relative risk estimates [26–30].

Ecological methods, when applied to assessment of a signal association with a targeted vaccination campaign and a disease with a potentially long interval from onset to diagnosis, can provide an unbiased estimate of vaccine-associated risk in a very limited set of circumstances. Obtaining an unbiased estimate in the absence of an association is possible even with very low vaccine coverage and a long onset to diagnosis interval. However, in the presence of a true vaccine-associated risk, all estimates will be biased toward one; this bias is reduced when cases are detected quickly and vaccination coverage is high. Based upon simulations, the estimates we obtained in the current study appear to be underestimations of true relative risks greater than one. However, the simulations did not take into account the possibility of increased reporting due to an awareness of the association, which has been shown in previous simulations to inflate risk estimates [8].

The predicted underlying individual-level relative risk obtained using models derived from simulated data, given the low vaccine coverage attained in most sites, are remarkably high. It is very unlikely that the true relative risk in Sweden, for example, is 36-fold. Calibrated estimates for The United Kingdom, however, are only slightly lower than those reported by Miller et al in their study of narcolepsy following Pandemrix^TM^ vaccination in children aged 4–18 [31]. In general, these predicted relative risks are not in line with results found in the case-control study conducted within SOMNIA, in which no increased risk following pH1N1 vaccines was detected [32, 33]. It is important to note that while the case-control data in the SOMNIA study did not include any pediatric cases exposed to Pandemrix^TM^, the case-coverage sub-study of pediatric cases in The Netherlands did not find an association with Pandemrix^TM^ [33]. These inconsistencies may be an illustration of the ecological fallacy, namely that associations detected at the population level may not be causal at the individual level [34]. In fact, as coverage in our simulations approached 100%, our population-level analysis also approached an individual-level analysis with accurate exposure data for all subjects, explaining why increased vaccine coverage in simulations leads to more accurate estimates of the simulated relative risk.

Previous simulations have shown that reduction in the time from onset to diagnosis following awareness of an association increase risk estimates [8]. Our simulation did not take into account factors that may have changed over the course of the study period such as awareness of narcolepsy and of the pH1N1-narcolepsy association as well as changes in diagnostic and coding practices. Each of these likely contributed to the IRR estimates we obtained. What our simulations do show is that in the absence of factors that increase case detection in the post-exposure period, detection of increased population-level risk of a disease with a long onset to diagnosis interval using ecological methods requires an extreme underlying individual-level risk.

The ecological approach fails to detect any increased risk unless the time from onset to diagnosis is short and both coverage and the true relative risk are high. Because of this, we recommend that population-level methods be used in assessment of outcomes with a delay from onset to diagnosis only to generate hypotheses or to strengthen signals when population-level exposure is high. Analysis of the full population when increased risk is only present in one age stratum performs as well as stratified analysis in terms of the magnitude of risk detected, but fails to identify the source of the increased risk. Therefore, if increased risk is suspected in a subset of the population, analyses should be stratified.

## Conclusions

Ecological methods can be useful in assessment of vaccine safety but it is important for investigators to understand the impacts of masking by strata not at risk, patterns of onset and diagnosis, and vaccine coverage. What appears to be an estimate of no effect could be valid or, as shown in our simulations, could be an underestimation.

## Supporting information

S1 Table Characteristics of the Databases in this study. * Linked Medical Records = Population based medical records (GP and specialist diagnoses), directly linked; Population-based registry = Population based registries (emergency room, in and out patient diagnoses); Medical Record diagnoses + Census Population = In and outpatient diagnoses, case counts and population counts (census);
1. http://www.epic-uk.org/our-data/our-data.shtml
2. http://www.erasmusmc.nl/med_informatica/research/555688/?lang=en#
3. http://www.kea.au.dk/en/ResearchRegistries.html
4. http://www.sidiap.org/index.php/en
5. http://fisabio.san.gva.es/en/fisabio;jsessionid=AFE38E9ACF0A380A692A9739E88F2FF4
6. http://www.socialstyrelsen.se/english
7. http://www.mohw.gov.tw/CHT/DOS/DM1.aspx?f_list_no=812 (Chinese)
8. http://www.health.alberta.ca/documents/Research-Health-Datasets.pdf
9. http://umanitoba.ca/faculties/health_sciences/medicine/units/chs/departmental_units/mchp/resources/repository/index.html
10. https://www.popdata.bc.ca/data.
(DOCX)

S2 Table Incidence Rates and Incidence Rate Ratios by continent, country, age, and period. *Periods are as follows: Pre-Circulation = January 2003-the beginning of wild-type H1N1 circulation (defined per country); Circulation = Period from the beginning of wild-type H1N1 circulation until the start of the vaccination campaign (defined per country); Vaccination & Post = Period from the beginning of the vaccination campaign through December 2013.
† IRR comparing the period to the pre-circulation period, within the age group
‡The count has been suppressed either because (1) the observed number of events is very small (n ≤ 2) and not appropriate for publication; or (2) it could be used to calculate the number in a cell that has been suppressed.
(DOCX)

S3 Table IRRs of Post-Vaccination vs. Pre-Circulation in categories of Coverage and Adjuvant. (DOCX)
