# Supplementary material for: Incidence rates of narcolepsy diagnoses in Taiwan, Canada, and Europe: The use of statistical simulation to evaluate methods for the rapid assessment of potential safety issues on a population level in the SOMNIA study
Source: PLoS One. 2018 Oct 17;13(10):e0204799. doi: 10.1371/journal.pone.0204799 (PMC6192586; doi:10.1371/journal.pone.0204799)
Supplement: S1 Table — * Linked Medical Records = Population based medical records (GP and specialist diagnoses), directly linked; Population-based registry = Population based registries (emergency room, in and out patient diagnoses); Medical Record diagnoses + Census Population = In and outpatient diagnoses, case counts and population counts (census); 1. http://www.epic-uk.org/our-data/our-data.shtml 2. http://www.erasmusmc.nl/med_informatica/research/555688/?lang=en# 3. http://www.kea.au.dk/en/ResearchRegistries.html 4. http://www.sidiap.org/index.php/en 5. http://fisabio.san.gva.es/en/fisabio;jsessionid=AFE38E9ACF0A380A692A9739E88F2FF4 6. http://www.socialstyrelsen.se/english 7. http://www.mohw.gov.tw/CHT/DOS/DM1.aspx?f_list_no=812 (Chinese) 8. http://www.health.alberta.ca/documents/Research-Health-Datasets.pdf 9. http://umanitoba.ca/faculties/health_sciences/medicine/units/chs/departmental_units/mchp/resources/repository/index.html 10. https://www.popdata.bc.ca/data. (DOCX) [file pone.0204799.s001.docx]

**Supplementary Table 1: Characteristics of the Databases in this study**

| Data site & source | Type of data* | Algorithm used | H1N1 virus circulation (weeks) | Vaccination Coverage by age group | Adjuvant Used by age group | |
| --- | --- | --- | --- | --- | --- | --- |
| UK THIN^1^ | Linked Medical Records | Read codes F27.00, F270.00, F271.00, F27z.00 | 2009: 26-52 | 6 mo-5yr (20%)  5-18 yr (4%)  >65yr (35%) | 05-19: AS03 (Pandemrix)  20-59: AS03 (Pandemrix) | |
| NL IPCI^2^ | Linked Medical Records | Free text narcolepsy & MSLT. Followed by manual review | 2009: 30-50 | < 5yr (75%)  Risk groups (70%) | 05-19: MF59  20-59: MF59 | |
| DK AARHUS^3^ | Population-based registry | ICD-10 code G47.4 (primary and secondary) diagnosis | 2009: 29-45 | >18yr (20%) | 05-19: AS03 (Pandemrix)  20-59: AS03 | |
| SIDIAP  (Spain, Catalunya)^4^ | Linked Medical Records | ICD-10 code G47.4 diagnosis | 2009: 31-50 | < 18yr (1%)  all population (3.5%) | 05-19: MF59  20-59: AS03 (Pandemrix) | |
| FISABIO  (Spain, Valencia)^5^ | Linked Medical Records | ICD-9CM codes 347.* with Manual validation | 2009: 31-50 | 6mo-14yr, risk groups (11%)  15-59yr, risk groups (13%)  >60yr, risk groups (28%)  Pregnant women (9%)  Healthcare workers (30%) | 05-19: MF59  20-59: AS03 (Pandemrix) | |
| Sweden^6^ | Medical Record diagnoses + Census Population | ICD-10 code G47.4 diagnosis | 2009: 30-50 | \| < 18 yr (12%) \| \| --- \| \| > 18 yr (13%) \| | 05-19: AS03 (Pandemrix)  20-59: AS03 (Pandemrix) | |
| Taiwan^7^ | Linked Medical Records | ICD9-CM codes 347.* with MSLT procedure | 2009: 30-52 | 6mo-18yr (59%)  > 19yr (11%) | | 05-19: MF59  20-59: MF59 |
| Canada, Alberta^8^ | Linked Medical Records | ICD9-CM codes 347.* with MSLT procedure | 2009: 19-27 | ≥12 yr (37%) | | 05-19: AS03 (Arepanrix)  20-59: AS03 |
| Canada, Manitoba^9^ | Linked Medical Records | ICD9-CM codes 347.* with MSLT procedure | 2009: 19-27 | ≥12 yr (37%) | | 05-19: AS03 (Arepanrix)  20-59: AS03 |
| Canada, British Columbia^10^ | Medical Record diagnoses + Census Population | ICD9-CM codes 347.* with MSLT procedure | 2009: 19-27 | <10yr (46%)  10-18yr (32%)  19-39yr (33%)  40-64yr (45%)  >64 (58%) | | 05-19: AS03 (Arepanrix)  20-59: AS03 |

* Linked Medical Records = Population based medical records (GP and specialist diagnoses), directly linked; Population-based registry = Population based registries (emergency room, in and out patient diagnoses); Medical Record diagnoses + Census Population = In and outpatient diagnoses, case counts and population counts (census);

1. <http://www.epic-uk.org/our-data/our-data.shtml>
2. [http://www.erasmusmc.nl/med_informatica/research/555688/?lang=en#](http://www.erasmusmc.nl/med_informatica/research/555688/?lang=en)
3. <http://www.kea.au.dk/en/ResearchRegistries.html>
4. <http://www.sidiap.org/index.php/en>
5. <http://fisabio.san.gva.es/en/fisabio;jsessionid=AFE38E9ACF0A380A692A9739E88F2FF4>
6. <http://www.socialstyrelsen.se/english>
7. <http://www.mohw.gov.tw/CHT/DOS/DM1.aspx?f_list_no=812> (Chinese)
8. <http://www.health.alberta.ca/documents/Research-Health-Datasets.pdf>
9. <http://umanitoba.ca/faculties/health_sciences/medicine/units/chs/departmental_units/mchp/resources/repository/index.html>
10. <https://www.popdata.bc.ca/data>
