# Supplementary material for: Incidence rates of narcolepsy diagnoses in Taiwan, Canada, and Europe: The use of statistical simulation to evaluate methods for the rapid assessment of potential safety issues on a population level in the SOMNIA study
Source: PLoS One. 2018 Oct 17;13(10):e0204799. doi: 10.1371/journal.pone.0204799 (PMC6192586; doi:10.1371/journal.pone.0204799)
Supplement: S2 Table — *Periods are as follows: Pre-Circulation = January 2003-the beginning of wild-type H1N1 circulation (defined per country); Circulation = Period from the beginning of wild-type H1N1 circulation until the start of the vaccination campaign (defined per country); Vaccination & Post = Period from the beginning of the vaccination campaign through December 2013. † IRR comparing the period to the pre-circulation period, within the age group ‡The count has been suppressed either because (1) the observed number of events is very small (n ≤ 2) and not appropriate for publication; or (2) it could be used to calculate the number in a cell that has been suppressed. (DOCX) [file pone.0204799.s002.docx]

**Supplementary Table 2: Incidence rates and Incidence Rate Ratios by continent, country, age and period**

| Continent | Site | Age | Period* | Cases | Person  years | IR | IRR† | 95%CI |
| --- | --- | --- | --- | --- | --- | --- | --- | --- |
| Europe | United Kingdom | 0-4 | Pre-Circulation | 0 | 1,438,688 | 0.00 | Ref | -- |
|  |  |  | Circulation | 0 | 82033 | 0.00 | NA | -- |
|  |  |  | Vaccination & Post | 1 | 986145 | 0.10 | NA | -- |
|  |  | 5-19 | Pre-Circulation | 26 | 4247239 | 0.61 | Ref | -- |
|  |  |  | Circulation | 0 | 229303 | 0.00 | NA | -- |
|  |  |  | Vaccination & Post | 28 | 2752486 | 1.02 | 1.66 | 0.97, 2.83 |
|  |  | 20-59 | Pre-Circulation | 183 | 13782669 | 1.33 | Ref | -- |
|  |  |  | Circulation | 9 | 744620 | 1.21 | 0.91 | 0.47, 1.78 |
|  |  |  | Vaccination & Post | 90 | 8706262 | 1.03 | 0.78 | 0.61, 1.00 |
|  |  | 60+ | Pre-Circulation | 84 | 5738919 | 1.46 | Ref | -- |
|  |  |  | Circulation | 8 | 331682 | 2.41 | 1.65 | 0.80, 3.40 |
|  |  |  | Vaccination & Post | 38 | 3857675 | 0.99 | 0.67 | 0.46, 0.99 |
|  | Denmark | 0-4 | Pre-Circulation | 0 | 625074 | 0.00 | Ref | -- |
|  |  |  | Circulation | 0 | 51527 | 0.00 | NA | -- |
|  |  |  | Vaccination & Post | 2 | 426447 | 0.47 | NA | -- |
|  |  | 5-19 | Pre-Circulation | 26 | 1941950 | 1.34 | Ref | -- |
|  |  |  | Circulation | 3 | 160562 | 1.87 | 1.40 | 0.42, 4.61 |
|  |  |  | Vaccination & Post | 28 | 1352428 | 2.07 | 1.55 | 0.91, 2.64 |
|  |  | 20-59 | Pre-Circulation | 103 | 5258884 | 1.96 | Ref | -- |
|  |  |  | Circulation | 8 | 416864 | 1.92 | 0.98 | 0.48, 2.01 |
|  |  |  | Vaccination & Post | 58 | 3492758 | 1.66 | 0.85 | 0.62, 1.17 |
|  |  | 60+ | Pre-Circulation | 25 | 2189220 | 1.14 | Ref | -- |
|  |  |  | Circulation | 3 | 195788 | 1.53 | 1.34 | 0.41, 4.44 |
|  |  |  | Vaccination & Post | 13 | 1738626 | 0.75 | 0.66 | 0.34, 1.28 |
|  | The Netherlands | 0-4 | Pre-Circulation | 0 | 41272 | 0.00 | Ref | -- |
|  |  |  | Circulation | 0 | 10442 | 0.00 | NA | -- |
|  |  |  | Vaccination & Post | 0 | 129291 | 0.00 | NA | -- |
|  |  | 5-19 | Pre-Circulation | 2 | 103950 | 1.92 | Ref |  |
|  |  |  | Circulation | 0 | 29453 | 0.00 | NA |  |
|  |  |  | Vaccination & Post | 1 | 394895 | 0.25 | 0.13 | 0.01, 1.45 |
|  |  | 20-59 | Pre-Circulation | 2 | 306773 | 0.65 | Ref |  |
|  |  |  | Circulation | 1 | 87315 | 1.15 | 1.76 | 0.16, 19.37 |
|  |  |  | Vaccination & Post | 7 | 1144346 | 0.61 | 0.94 | 0.20, 4.52 |
|  |  | 60+ | Pre-Circulation | 0 | 111122 | 0.00 | Ref | -- |
|  |  |  | Circulation | 0 | 35025 | 0.00 | NA | -- |
|  |  |  | Vaccination & Post | 1 | 488348 | 0.20 | NA | -- |
|  | Spain | 0-4 | Pre-Circulation | 0 | 755198 | 0.00 | Ref | -- |
|  |  |  | Circulation | 0 | 601869 | 0.00 | NA | -- |
|  |  |  | Vaccination & Post | 0 | 1647061 | 0.00 | NA | -- |
|  |  | 5-19 | Pre-Circulation | 7 | 1617473 | 0.43 | Ref |  |
|  |  |  | Circulation | 4 | 1488771 | 0.27 | 0.62 | 0.18, 1.13 |
|  |  |  | Vaccination & Post | 26 | 4715178 | 0.55 | 1.27 | 0.55, 2.94 |
|  |  | 20-59 | Pre-Circulation | 48 | 6847254 | 0.70 | Ref | -- |
|  |  |  | Circulation | 33 | 610444 | 0.54 | 0.77 | 0.50, 1.20 |
|  |  |  | Vaccination & Post | 125 | 18915104 | 0.27 | 0.94 | 0.68, 1.31 |
|  |  | 60+ | Pre-Circulation | 7 | 2728360 | 0.26 | Ref | -- |
|  |  |  | Circulation | 11 | 2372937 | 0.46 | 1.82 | 0.70, 4.76 |
|  |  |  | Vaccination & Post | 25 | 7526241 | 0.33 | 1.29 | 0.56, 2.99 |
|  | Sweden | 0-4 | Pre-Circulation | 13 | 3166715 | 0.41 | Ref | -- |
|  |  |  | Circulation | 4 | 274111 | 1.46 | 3.56 | 1.16, 10.90 |
|  |  |  | Vaccination & Post | 7 | 2418815 | 0.29 | 0.71 | 0.28, 1.77 |
|  |  | 5-19 | Pre-Circulation | 62 | 10381883 | 0.60 | Ref | -- |
|  |  |  | Circulation | 1 | 819877 | 0.12 | 0.20 | 0.03, 1.47 |
|  |  |  | Vaccination & Post | 369 | 6854603 | 5.38 | 9.01 | 6.89, 11.80 |
|  |  | 20-59 | Pre-Circulation | 338 | 29823712 | 1.13 | Ref | -- |
|  |  |  | Circulation | 26 | 2418238 | 1.08 | 0.95 | 0.64, 1.41 |
|  |  |  | Vaccination & Post | 401 | 20992445 | 1.91 | 1.69 | 1.46, 1.95 |
|  |  | 60+ | Pre-Circulation | 200 | 13550206 | 1.48 | Ref | -- |
|  |  |  | Circulation | 12 | 1158116 | 1.04 | 0.70 | 0.39, 1.26 |
|  |  |  | Vaccination & Post | 103 | 10168490 | 1.01 | 0.69 | 0.54, 0.87 |
| North America | Canada | 0-4 | Pre-Circulation | 1 | 2669395 | 0.04 | Ref | -- |
|  |  |  | Circulation | 0 | 346294 | 0.00 | NA | -- |
|  |  |  | Vaccination & Post | 3 | 2021834 | 0.15 | 3.96 | 0.41, 38.08 |
|  |  | 5-19 | Pre-Circulation | 67 | 10107116 | 0.66 | Ref | -- |
|  |  |  | Circulation | 6 | 1261204 | 0.48 | 0.72 | 0.31, 1.70 |
|  |  |  | Vaccination & Post | 53 | 6378494 | 0.83 | 1.25 | 0.87, 1.80 |
|  |  | 20-59 | Pre-Circulation | 265 | 34413993 | 0.77 | Ref | -- |
|  |  |  | Circulation | 36 | 4574717 | 0.79 | 1.02 | 0.72, 1.45 |
|  |  |  | Vaccination & Post | 182 | 24228401 | 0.75 | 0.98 | 0.81, 1.18 |
|  |  | 60+ | Pre-Circulation | 77 | 10259739 | 0.75 | Ref | -- |
|  |  |  | Circulation | 8 | 1481882 | 0.54 | 0.72 | 0.35, 1.49 |
|  |  |  | Vaccination & Post | 49 | 8335817 | 0.59 | 0.78 | 0.55, 1.12 |
| Asia | Taiwan | 0-4 | Pre-Circulation | 0 | 3647009 | 0.00 | Ref | -- |
|  |  |  | Circulation | 0 | 256532 | 0.00 | NA | -- |
|  |  |  | Vaccination & Post | 0 | 2526902 | 0.00 | NA | -- |
|  |  | 5-19 | Pre-Circulation | 81 | 13985353 | 0.58 | Ref | -- |
|  |  |  | Circulation | 16 | 1103680 | 1.45 | 2.50 | 1.46, 4.28 |
|  |  |  | Vaccination & Post | 110 | 11867183 | 0.93 | 1.60 | 1.20, 2.13 |
|  |  | 20-59 | Pre-Circulation | 78 | 46806947 | 0.17 | Ref | -- |
|  |  |  | Circulation | 14 | 3768896 | 0.37 | 2.23 | 1.26, 3.94 |
|  |  |  | Vaccination & Post | 158 | 44542437 | 0.35 | 2.13 | 1.62, 2.79 |
|  |  | 60+ | Pre-Circulation | 8 | 17512300 | 0.05 | Ref | -- |
|  |  |  | Circulation | ‡ | ‡ | 0.08 | 1.72 | 0.22, 13.76 |
|  |  |  | Vaccination & Post | ‡ | ‡ | 0.04 | 0.93 | 0.32, 2.68 |

*Periods are as follows: Pre-Circulation = January 2003-the beginning of wild-type H1N1 circulation (defined per country); Circulation = Period from the beginning of wild-type H1N1 circulation until the start of the vaccination campaign (defined per country); Vaccination & Post = Period from the beginning of the vaccination campaign through December 2013.

† IRR comparing the period to the pre-circulation period, within the age group

‡The count has been suppressed either because (1) the observed number of events is very small (n ≤ 2) and not appropriate for publication; or (2) it could be used to calculate the number in a cell that has been suppressed.
