# Supplementary material for: Incidence rates of narcolepsy diagnoses in Taiwan, Canada, and Europe: The use of statistical simulation to evaluate methods for the rapid assessment of potential safety issues on a population level in the SOMNIA study
Source: PLoS One. 2018 Oct 17;13(10):e0204799. doi: 10.1371/journal.pone.0204799 (PMC6192586; doi:10.1371/journal.pone.0204799)
Supplement: S3 Table — (DOCX) [file pone.0204799.s003.docx]

**Supplementary Table 3: IRRs Post-Vaccination vs. Pre-Circulation in categories of Coverage and Adjuvant**

| Age Group | Adjuvant | Coverage | Countries | IRR | 95% CI |
| --- | --- | --- | --- | --- | --- |
| Children (5-19 years) | MF59 | Low | Netherlands  Spain | 1.01 | 0.45, 2.25 |
|  | AS03 | Low | Denmark  United Kingdom | 1.62 | 1.09, 2.42 |
|  | AS03 | High | Sweden | 9.01 | 6.78, 11.99 |
| Adults (20-59 years) | MF59 | Low | Netherlands | 0.94 | 0.18, 4.97 |
|  | AS03 | Low | Spain  Denmark  United Kingdom | 0.68 | 0.57, 0.81 |
|  | AS03 | High | Sweden | 1.69 | 1.45, 1.97 |
